# fMRI replicability depends upon sufficient individual-level data

**DOI:** 10.1038/s42003-019-0378-6

**Published:** 2019-04-12

**Authors:** Derek Evan Nee

**Affiliations:** Department of Psychology and College of Medicine, Florida State University, Tallahassee, FL 32306-4301 USA

**Arising from** B.O. Turner et al. *Communications Biology* 10.1038/s42003-018-0073-z (2018)

The reproducibility of task-based functional magnetic resonance imaging (fMRI), or lack thereof, has become a topic of intense scrutiny^1,2^. Relative to other human techniques, fMRI has high costs associated with data collection, storage, and processing. To justify these costs, the inferences gained from fMRI need to be robust and meaningful. Hence, although large, sufficiently powered data sets may be costly, this is favorable to collecting many insufficiently powered data sets from which reliable conclusions cannot be drawn. However, it can be difficult to determine a priori how much data are needed. Although power analyses can help^3^, accurately calculating power itself requires an appropriate estimate of the expected effect size, which can be hard to obtain if previous studies had insufficient data to produce reliable effect size estimates. Furthermore, mechanistic basic science explores novel phenomena with innovative paradigms such that extrapolation of effect sizes from existing data may not be appropriate.

In light of these issues, many studies rely on rules-of-thumb to determine the amount of data to be collected. For example, Thirion et al.^4^ suggested that 20 or more participants are required for reliable task-based fMRI inferences. Turner et al.^5^ recently pointed out that such recommendations are outdated, and set out to empirically estimate replicability using large data sets. The authors found that even data sets with 100 or more participants can produce results that do not replicate, suggesting that large sample sizes are necessary for task-based fMRI.

It is typical for considerations of power in task-based fMRI to focus on sample size. This is because between-subject variability tends to dominate within-subject variability, such that sampling more subjects is often a more effective use of time than scanning individuals for longer^3,4^. Large task-based fMRI data collections such as the Human Connectome Project (HCP) have used batteries of tasks wherein each task is scanned on the order of 10 min^6^. Such batteries operate under the assumption that within-subject variability, which diminishes with scan time, can reach appropriately low levels within a relatively short period. However, using data from the HCP and other data of similar durations, Turner et al.^5^ demonstrated that task-based fMRI can be unreliable.

With the rising popularity of resting-state fMRI, investigators have examined the duration of resting-state data needed for reliable parameter estimates. Some have suggested that parameter estimates are stable after 5–10 min of resting-state scans^7^, although more recent data suggest 30–40 min are needed^8,9^. In either case, parameters estimated from rest use the entire (cleaned) data time-series, while task-based fMRI splits the time-series into composite mental events. For example, in a rapid event-related design, there may be ~4–6 s of peak signal attributable to a given transient event-of-interest (e.g., a choice reaction). If 20 such events exist in a 10-minute task run, that amounts to less than < 2 min of signal attributable to that task event. Although it is difficult to extrapolate from rest to task given the numerous differences between the methods, it is likely that parameter estimates in such short tasks would benefit from additional measurements at the individual-level.

To examine the impact of individual-level measurements on task-based fMRI replicability, I re-analyzed data from a recently published pair of data sets^10,11^. Each data set estimated five contrasts-of-interest spanning main effects and an interaction in a 2 × 2 × 2 factorial design. The resultant contrasts variously load on often-studied constructs of working memory, task-switching, language, and spatial attention. These constructs have a high degree of overlap with those examined by Turner et al.^5^ Previously, I suggested the reproducibility in these data were good^10,11^, but given the observations of Turner et al.^5^, the sample sizes employed (*n* = 24) should produce low replicability. On the other hand, ~1–2 hours of task data were collected for each individual, which could have facilitated reliability. To formally examine this matter, I computed the replicability measures of Turner et al.^5^ on randomly sub-sampled independent data sets for the five contrasts-of-interest. I varied the amounts of individual-level data from ~10 minutes (one task run) to ~1 hour (six task runs). I also varied the sample size from 16 to 23 individuals with 16 matching the minimum examined by Turner et al.^5^ and 23 being the maximum that can be split into independent groups in the 46 participants examined. All data and code are available at https://osf.io/b7y9n.

Figure 1 shows the results at *n* = 16. When only one run is included for each individual, the replicability estimates fall in the ranges reported by Turner et al.^5^. However, reproducibility markedly improved with more data at the individual-level. Although there are some indications of diminishing returns after four runs, there were clear benefits to more scans at the individual-level. Figure 2 reports the results at *n* = 23, which again show clear benefits to reproducibility with >1 run. For example, the mean peak replicability with two runs (~ 65%) matches observations in Turner et al.^5^ at *n* = 64. Furthermore, no contrast in Turner et al.^5^ approached perfect replicability with any combination of measure, sample size, and threshold, whereas multiple combinations produced near perfect replicability for the Contextual Control contrast with as little as six runs at *n* = 16 (Supplemental Fig. 1). In the most striking such case, I find ~ 90% of the peaks replicate on average with four runs at *n* = 23 (Supplemental Fig. 2), which again exceeded the observations of Turner et al.^5^ even at the largest sample size (*n* = 121). Although the differences in tasks employed here and those in Turner et al.^5^ qualify direct comparisons, the data here paint a much more reliable picture of task-based fMRI at modest sample sizes when individuals are adequately sampled.

**Fig. 1:**
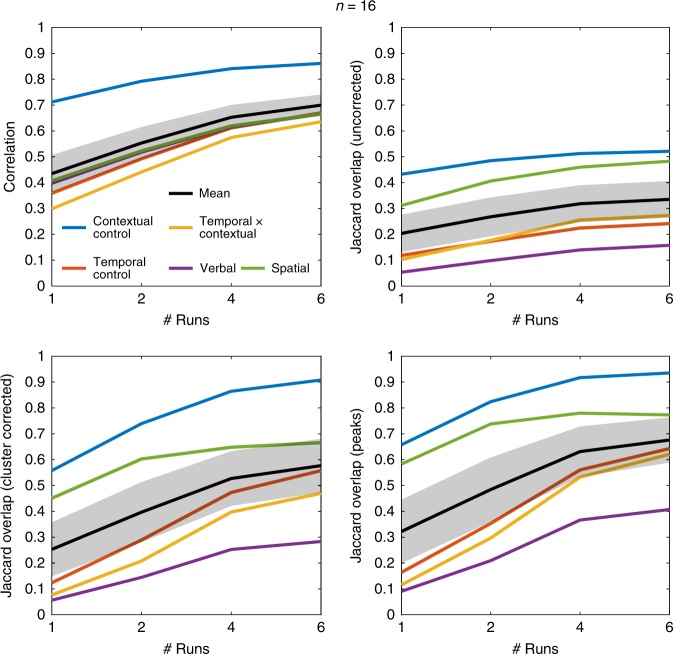
Replicability estimates at *n* = 16. Metrics correspond to those used in Turner et al.^5^. Jaccard Overlaps were calculated using conservative thresholds comparable to those reported in Turner et al.^5^. Error bands represent one standard error of the mean

**Fig. 2:**
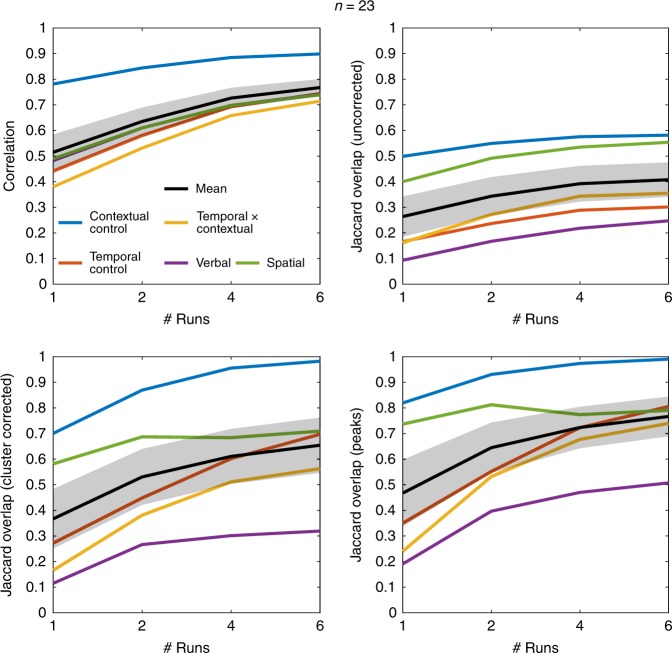
Replicability estimates at *n* = 23. Other details match Fig. 1

These observations raise the question of how much individual-level data are needed. This is not straightforward to determine a priori and hinges on the ratio of within- to between-subject variability and effect magnitude (see ref. ^12^ for demonstrations of how these factors trade-off). Concrete recommendations are rendered difficult given that these factors will vary considerably based on experimental design (including how the data are modeled), brain region, population, scanner, and scanning parameters. In the data explored here, at *n* = 23 with six runs, peaks from the Contextual Control contrast were nearly perfectly reliable, although only half of the peaks from the Verbal contrast replicated despite these contrasts being matched for time and number of trials, demonstrating that one size does not fit all. In general, more data at the individual level are beneficial when within-subject variability is high, and between-subject variability is low^12^. Furthermore, across all of the contrasts, I observed diminishing returns after approximately four task runs, which may owe to the duration of time participants can remain attentive and still (i.e., ~40 minutes) and/or the point at which the within-subject variability is sufficiently low relative to the between-subject variability. Hence, 40 minutes of task may be a reasonable starting point for pilot data, from which the appropriate parameters can be estimated and used to determine proper levels of *n* and scan time.

A final question is the extent to which researchers are scanning sufficiently at the individual-level. An assay of recent studies of basic mechanistic research indicates that modest sample sizes are the norm (mean *N* = 31.7), but few studies employ less than ten-minute scanning durations (Supplemental Fig. 3). The average per task scanning duration was ~40 minutes, which matches the point of diminishing returns observed here. Hence, the observations of Turner et al.^5^ based on short scans cannot be broadly generalized to basic science research that tends to scan much longer. However, those studies employing batteries of short tasks would do well to consider the observations of Turner et al.^5^ and here, and collect more individual-level data to foster reproducibility.

## Methods

Full details of the participants, task, preprocessing, and modeling can be found in my previous reports^10,11^. In brief, the task manipulated two forms of cognitive control (contextual control, temporal control) and stimulus domain (verbal, spatial) in a 2 × 2 × 2 factorial design. Five contrasts from the factorial design were included in this report: contextual control, temporal control, temporal control×contextual control, verbal (> spatial), and spatial (> verbal). On each block, participants performed a sequence-matching task in a given stimulus domain. Then, sub-task phases orthogonally manipulated the cognitive control demands. In the original report, we examined stimulus domain (verbal > spatial, spatial > verbal) across all trials. But here, I use only the sub-task phases so that all contrasts have the same amount of data at the individual level. A separate contrast estimate was created for each individual and each run. I included data from 46 participants, excluding participants in the original reports that did not complete all of the task runs. Twenty-three participants performed 12 scanning runs and 23 participants performed 6 scanning runs, wherein each scanning run took ~ 10 min to complete. In both studies, informed consent was obtained in accordance with the Committee for Protection of Human Subjects at the University of California, Berkeley. Data and code are available at https://osf.io/b7y9n.

Following the procedures of Turner et al.^5^, replicability was determined by pairwise comparison of group-level t-statistic maps. For each analysis, the data were randomly split into two independent groups 500 times. Analyses varied the number of runs included at the individual level (1, 2, 4, or 6) by randomly selecting a subset of the data, and also the number of individuals (16 or 23). Extra-cranial voxels were masked out and voxels for which t-statistics could not be computed (i.e., owing to insufficient signal across participants) were discarded prior to computations of replicability.

The first analysis examined the voxel-wise correlation of t-statistics across all voxels. Subsequent analyses examined Jaccard overlap on thresholded t-statistic maps where the Jaccard overlap indicates the proportion of results that replicate. Although Turner et al.^5^ utilized both positive and negative activations for their Jaccard overlap calculations, here I use only positive activations given that two of the contrasts are the inverses of one another. Following Turner et al.^5^, Jaccard overlap was computed at the voxel-level by first thresholding the complete group data set and determining the number of significant voxels, *v*, at a voxel-wise threshold. This map represented the “ground truth.” Then, in each pair of sub-sampled data sets, the conjunction of the top *v* voxels was divided by their union to determine the proportion of replicated voxels.

The voxel-level procedure does not attempt to control false-positives for each group analysis. Therefore, low replicability in this measure might be anticipated by the inclusion of false-positives. So, Turner et al.^5^ also performed family-wise error correction using cluster-level thresholding in each group map, and calculated the number of overlapping voxels passing correction. However, cluster-level correction allows for cluster-level, but not voxel-level inference. That is, the cluster is the unit of significance rather than the voxels within the cluster. Noting the number of overlapping voxels, therefore, does not capture the essence of whether a cluster has replicated or not. Therefore, I modified the procedure to determine the number of overlapping clusters rather than voxels. A cluster was deemed to have replicated if at least half of the voxels of that cluster were present in the replicate. Half is an arbitrary number intended to safeguard against trivial overlap. Finally, Turner et al.^5^ examined peak overlap determined by whether the peak of a given cluster was also significant in the replicate. This is likely to be an important practical metric of replicability given that replication attempts will often examine a small radius around the peak of a previous report.

As in Turner et al.^5^ each Jaccard overlap was performed at both a conservative threshold (depicted in the main text) and liberal threshold (depicted in the supplemental material). The liberal/conservative thresholds were as follows: voxel-level: *p* < 0.00025/0.00000025; cluster-level: *p* < 0.05 height, 1019 voxel extent/*p* < 0.01 height, 300 voxel extent, each achieving alpha < 0.01 according to 3dClustSim in AFNI. Interestingly, although it has been reported that liberal cluster-forming thresholds have inflated false-positives^13^, which would be expected to harm replicability, replicability measures improved at the more liberal thresholds, which was also observed in Turner et al.^5^ to some extent.

To quantify whether short or long scanning durations per task are the norm for the basic science domain from which the observed study is drawn, I searched PubMed for papers published since the start of 2015 using the terms “fMRI AND (cognitive control OR working memory)”. I excluded studies of special populations (e.g., patients, children) and interventional studies (e.g., drug, training) to focus on basic mechanistic research. The duration that each task was scanned was estimated from the reports. Functional localizer tasks producing regions-of-interest for a main task were excluded. The durations of the 244 resulting tasks are summarized in Supplemental Figure 3. The database is included at https://osf.io/b7y9n.

### Reporting Summary

Further information on experimental design is available in the Nature Research Reporting Summary linked to this article.

## Supplementary information

Supplemental Material

Reporting Summary

## Data Availability

Data needed to reproduce all reported findings are available at https://osf.io/b7y9n.

## Code availability

The code needed to reproduce all reported findings is available at https://osf.io/b7y9n.

## References

[1] Button KS (2013). Power failure: why small sample size undermines the reliability of neuroscience. Nat. Rev. Neurosci..

[2] Szucs D, Ioannidis JPA (2017). Empirical assessment of published effect sizes and power in the recent cognitive neuroscience and psychology literature. PLoS Biol..

[3] Mumford JA, Nichols TE (2008). Power calculation for group fMRI studies accounting for arbitrary design and temporal autocorrelation. Neuroimage.

[4] Thirion B (2007). Analysis of a large fMRI cohort: Statistical and methodological issues for group analyses. Neuroimage.

[5] Turner BO, Paul EJ, Miller MB, Barbey AK (2018). Small sample sizes reduce the replicability of task-based fMRI studies. Commun. Biol..

[6] Barch DM (2013). Function in the human connectome: task-fMRI and individual differences in behavior. Neuroimage.

[7] Van Dijk KRA (2010). Intrinsic functional connectivity as a tool for human connectomics: theory, properties, and optimization. J. Neurophysiol..

[8] Gordon EM (2017). Precision functional mapping of individual human brains. Neuron.

[9] Kong, R. et al. Spatial topography of individual-specific cortical networks predicts human cognition, personality, and emotion. *Cereb. Cortex*10.1093/cercor/bhy123 (2018). 10.1093/cercor/bhy123 PMC6519695 29878084

[10] Nee, D. E. & D’Esposito, M. The hierarchical organization of the lateral prefrontal cortex. *eLife* **5**, pii: e12112 (2016). 10.7554/eLife.12112 PMC4811776 26999822

[11] Nee, D. E. & D’Esposito, M. Causal evidence for lateral prefrontal cortex dynamics supporting cognitive control. *eLife* **6**, pii: e28040 (2017). 10.7554/eLife.28040 PMC5640427 28901287

[12] Desmond JE, Glover GH (2002). Estimating sample size in functional MRI (fMRI) neuroimaging studies: statistical power analyses. J. Neurosci. Methods.

[13] Eklund A, Nichols TE, Knutsson H (2016). Cluster failure: why fMRI inferences for spatial extent have inflated false-positive rates. Proc. Natl. Acad. Sci. USA.

