# Supplementary material for: fMRI replicability depends upon sufficient individual-level data
Source: Commun Biol. 2019 Apr 12;2:130. doi: 10.1038/s42003-019-0378-6 (PMC6461660; doi:10.1038/s42003-019-0378-6)
Supplement: Supplementary file 1 — Supplemental Material [file 42003_2019_378_MOESM1_ESM.pdf]

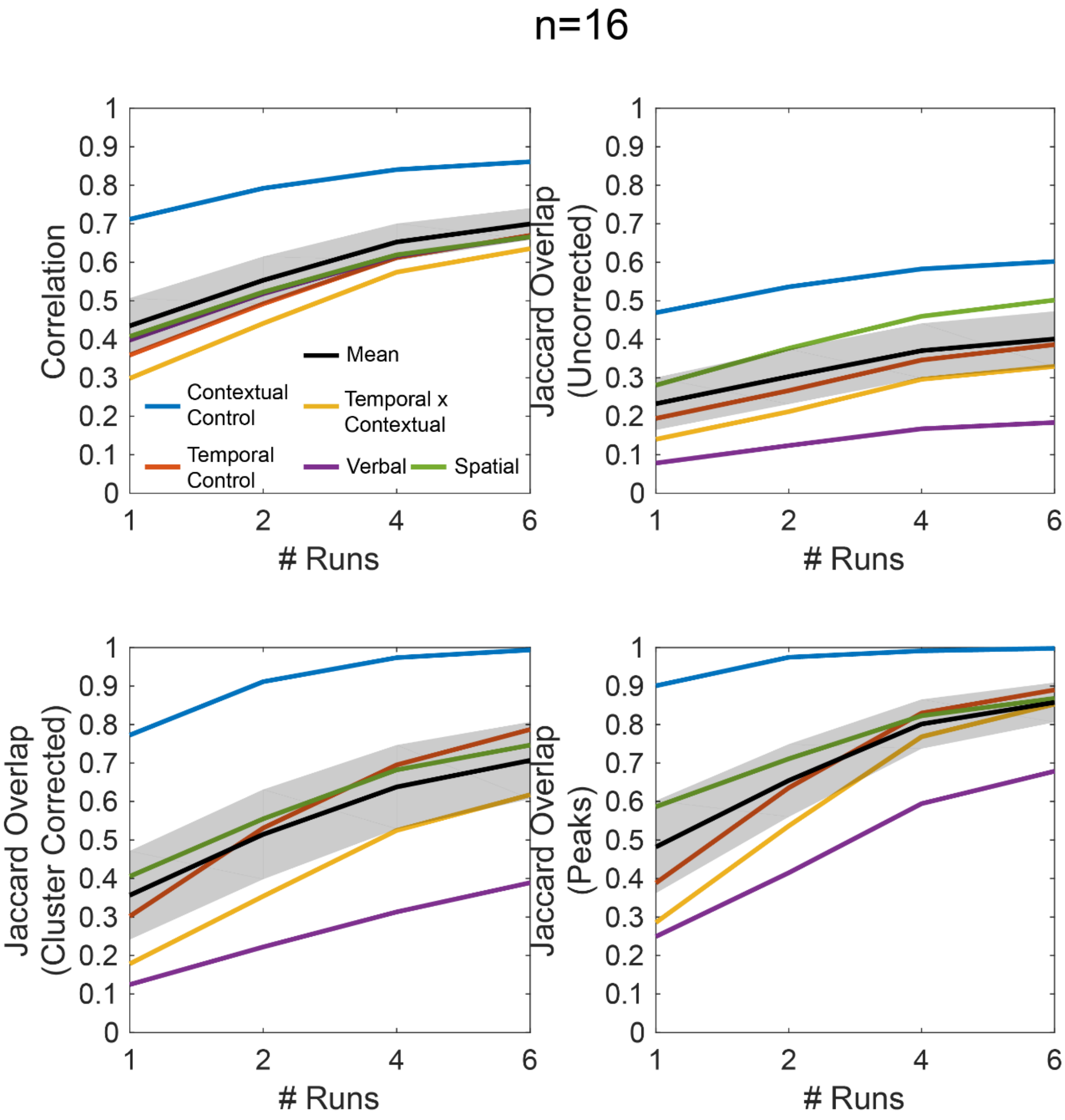

Supplemental Figure 1. Details are identical to Figure 1, but Jaccard Overlap was computed using liberal threshold comparable to those reported in Turner et al[1].

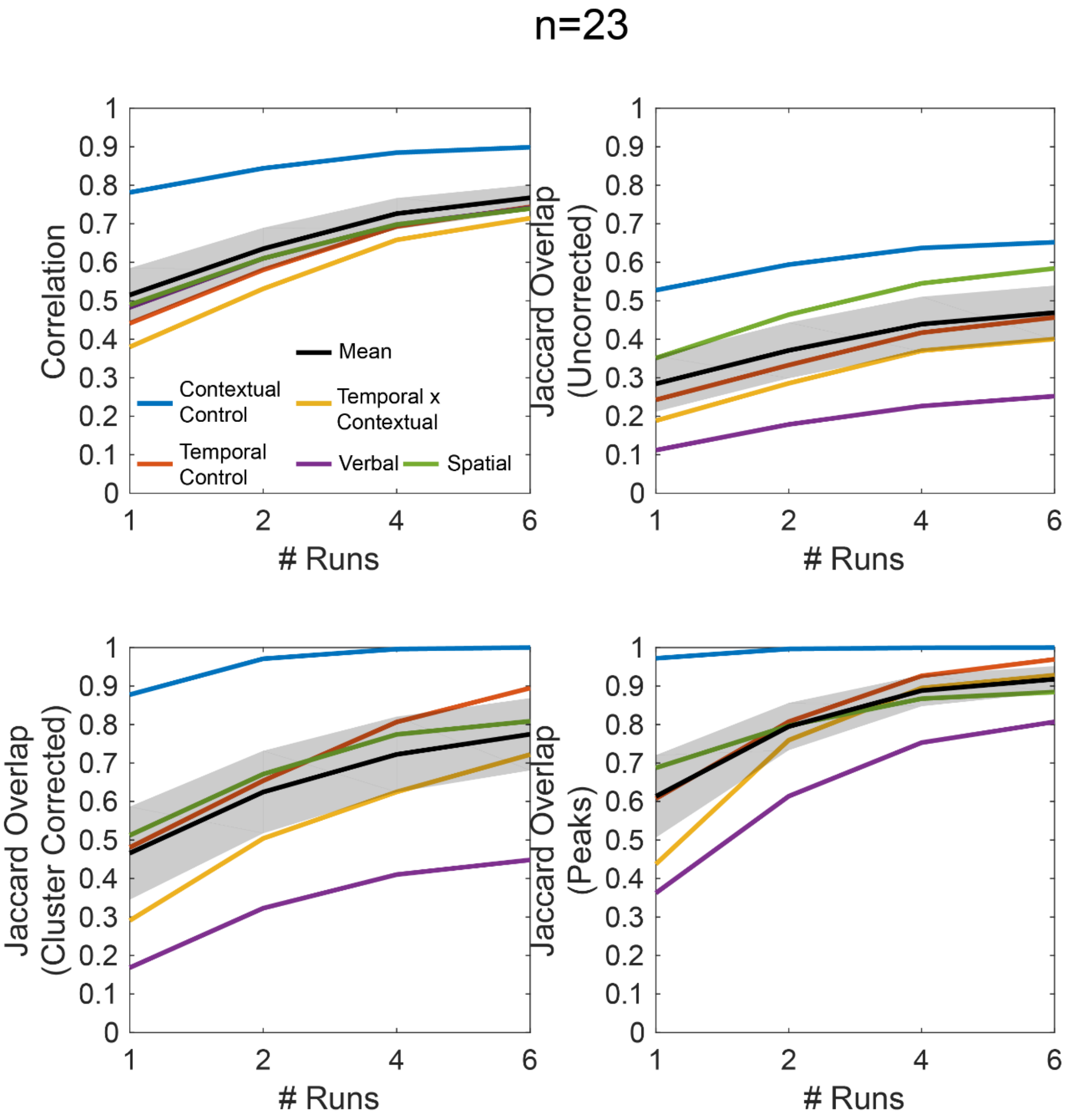

Supplemental Figure 2. Replicability estimates at n=23 with liberal thresholding. Other details match Supplemental Figure 1.

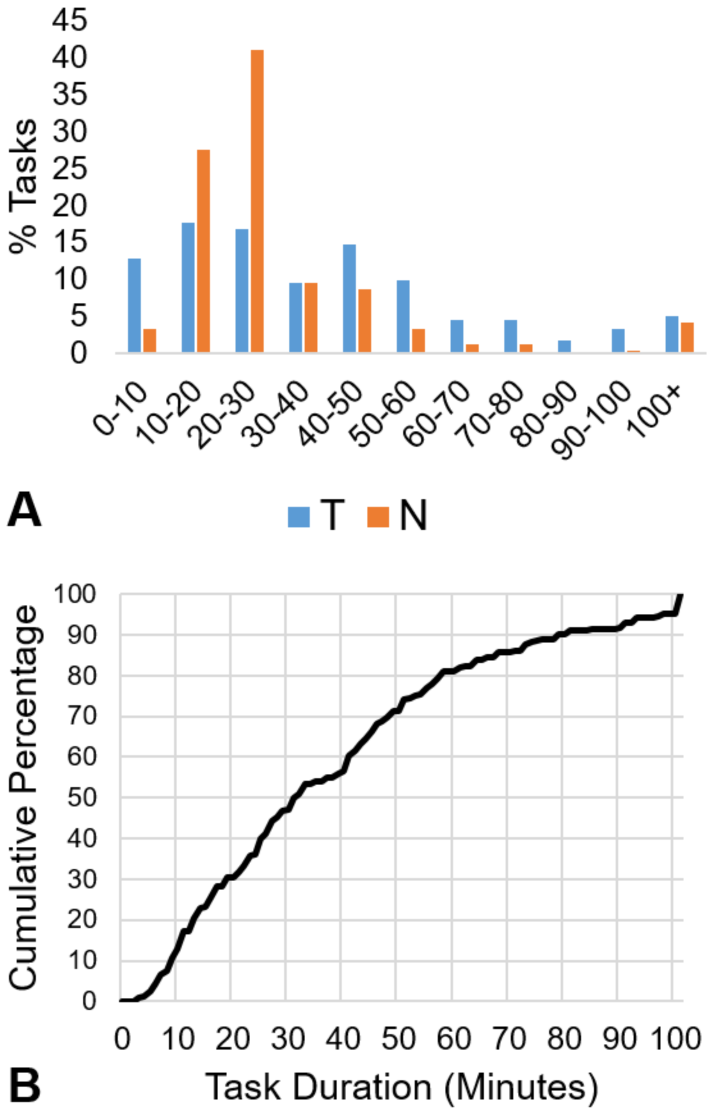

Supplemental Figure 3. Estimates of scanning durations and sample sizes for basic science research in the domains of cognitive control and working memory since 2015. A) Histograms of task durations in minutes (T) and sample size (N). Sample sizes tend to be modest (N=20-30), but task durations less than ten minutes were uncommon (<15%). Mean (standard deviation) of task duration was 39.86 (33.20) minutes, and sample size was 31.73 (34.88) participants. B) Cumulative percentage of task durations.

References

1. Turner, B. O., Paul, E. J., Miller, M. B. & Barbey, A. K. Small sample sizes reduce the replicability of task-based fMRI studies. *Commun. Biol.* **1**, (2018).
