# Supplementary material for: fMRI replicability depends upon sufficient individual-level data
Source: Commun Biol. 2019 Apr 12;2:130. doi: 10.1038/s42003-019-0378-6 (PMC6461660; doi:10.1038/s42003-019-0378-6)
Supplement: Supplementary file 2 — Reporting Summary [file 42003_2019_378_MOESM2_ESM.pdf]

nature research

Corresponding author(s): Derek Nee

Last updated by author(s): Jan 15, 2019

Reporting Summary

Nature Research wishes to improve the reproducibility of the work that we publish. This form provides structure for consistency and transparency in reporting. For further information on Nature Research policies, see Authors & Referees and the Editorial Policy Checklist.

Statistics

For all statistical analyses, confirm that the following items are present in the figure legend, table legend, main text, or Methods section.

n/a	Confirmed	
☐	☒	The exact sample size (n) for each experimental group/condition, given as a discrete number and unit of measurement
☐	☒	A statement on whether measurements were taken from distinct samples or whether the same sample was measured repeatedly
☐	☒	The statistical test(s) used AND whether they are one- or two-sided<br>*Only common tests should be described solely by name; describe more complex techniques in the Methods section.*
☒	☐	A description of all covariates tested
☐	☒	A description of any assumptions or corrections, such as tests of normality and adjustment for multiple comparisons
☐	☒	A full description of the statistical parameters including central tendency (e.g. means) or other basic estimates (e.g. regression coefficient) AND variation (e.g. standard deviation) or associated estimates of uncertainty (e.g. confidence intervals)
☒	☐	For null hypothesis testing, the test statistic (e.g. F, t, r) with confidence intervals, effect sizes, degrees of freedom and P value noted<br>*Give P values as exact values whenever suitable.*
☒	☐	For Bayesian analysis, information on the choice of priors and Markov chain Monte Carlo settings
☒	☐	For hierarchical and complex designs, identification of the appropriate level for tests and full reporting of outcomes
☒	☐	Estimates of effect sizes (e.g. Cohen's d, Pearson's r), indicating how they were calculated

Our web collection on statistics for biologists contains articles on many of the points above.

Software and code

Policy information about availability of computer code

Data collection	Stimuli were presented using E-Prime version 2.0
Data analysis	Data were analyzed using AFNI, SPM8, and custom MATLAB code

For manuscripts utilizing custom algorithms or software that are central to the research but not yet described in published literature, software must be made available to editors/reviewers. We strongly encourage code deposition in a community repository (e.g. GitHub). See the Nature Research guidelines for submitting code & software for further information.

Data

Policy information about availability of data

All manuscripts must include a data availability statement. This statement should provide the following information, where applicable:
- Accession codes, unique identifiers, or web links for publicly available datasets
- A list of figures that have associated raw data
- A description of any restrictions on data availability

All data and code are available at https://osf.io/b7y9n.

Field-specific reporting

Please select the one below that is the best fit for your research. If you are not sure, read the appropriate sections before making your selection.

☒ Life sciences ☐ Behavioural & social sciences ☐ Ecological, evolutionary & environmental sciences

For a reference copy of the document with all sections, see nature.com/documents/nr-reporting-summary-flat.pdf

Life sciences study design

All studies must disclose on these points even when the disclosure is negative.

Sample size	The targeted number of participants was based upon previous relevant literature. A sample size of 24 participants was targeted in each of the described studies as that number was within range of prior literature and allowed for perfect balancing of mappings described below.
Data exclusions	2 participants with incomplete data sets were excluded for this report.
Replication	Replication was the purpose of the study and all replication measures are described in detail in the manuscript.
Randomization	Shape-to-rule, color-to-feature, and decision-to-finger mappings were randomly counter-balanced.
Blinding	Blinding was not relevant given that all fMRI manipulations were repeated measures in a single experimental design.

Reporting for specific materials, systems and methods

We require information from authors about some types of materials, experimental systems and methods used in many studies. Here, indicate whether each material, system or method listed is relevant to your study. If you are not sure if a list item applies to your research, read the appropriate section before selecting a response.

Materials & experimental systems

n/a	Involved in the study	
☒	☐	Antibodies
☒	☐	Eukaryotic cell lines
☒	☐	Palaeontology
☒	☐	Animals and other organisms
☐	☒	Human research participants
☒	☐	Clinical data

Methods

n/a	Involved in the study	
☒	☐	ChIP-seq
☒	☐	Flow cytometry
☐	☒	MRI-based neuroimaging

Human research participants

Policy information about studies involving human research participants

Population characteristics	Healthy young adults ranging in age from 18-28 were included. Participants were right-handed native English speakers with no history of neurological or psychiatric disorders. 28 females and 20 males were included.
Recruitment	Recruitment was performed via online advertisements to the research subject volunteer pool listed in the University of California, Berkeley psychology website.
Ethics oversight	The protocol was approved by the Committee for Protection of Human Subjects at the University of California, Berkeley

Note that full information on the approval of the study protocol must also be provided in the manuscript.

Magnetic resonance imaging

Experimental design

Design type	Event-related
Design specifications	Half of the participants completed 1920 trials divided into 192 blocks with 24 blocks per cell of the 2 x 2 x 2 design. The other half of the participants completed 864 trials divided into 96 blocks with 12 blocks per cell of the 2 x 2 x 2 design.
Behavioral performance measures	Accuracy and reaction time were collected. Appropriate performance was verified by noting accuracies well above chance (50%) and accuracy and reaction time within the distribution of the group calculated via means and standard deviations.

Acquisition

Imaging type(s)	functional
Field strength	3 Tesla
Sequence & imaging parameters	EPI, 22.0 cm^2 FoV, 64x64 matrix, 3.8 mm slice thickness, 35 slices, TR = 2000 ms, TE = 25 ms, flip angle = 70 degrees
Area of acquisition	whole brain

Diffusion MRI ☐ Used ☒ Not used

Preprocessing

Preprocessing software	AFNI - 3dDespike, SPM8 - slice timing correction, realign and unwarp, segmented normalization, smoothing at 8 FWHM
Normalization	non-linear using a T1-weighted MPRAGE
Normalization template	ICBM152
Noise and artifact removal	For subjects with greater than 3mm total displacement or a framewise 0.5mm movement, motion regressors including linear, quadratic, differential, and quadratic differential terms were included as nuisance co-variates
Volume censoring	Data were visually inspected for artifacts. No individual volumes were censored.

Statistical modeling & inference

Model type and settings	Mass univariate with AR1 correction for auto-correlation at the first level. Random effects t-tests at the second level.
Effect(s) tested	Temporal control: (Dual+Delay)>(Restart+Control); Contextual control: (Dual+Restart)>(Delay+Control); Interaction: (Dual+Control)>(Delay+Restart); Spatial: Spatial>Verbal; Verbal: Verbal>Spatial

Specify type of analysis: ☒ Whole brain ☐ ROI-based ☐ Both

Statistic type for inference (See Eklund et al. 2016)	Cluster-wise at two levels: 0.05 height, 1019 voxel extent; 0.01 height, 300 voxel extent
Correction	FWE p < 0.01 based upon 3dClustStim from AFNI

Models & analysis

n/a	Involved in the study
☒	☐ Functional and/or effective connectivity
☒	☐ Graph analysis
☒	☐ Multivariate modeling or predictive analysis
